# Peroxisome Proliferator-Activated Receptor γ, but Not α or G-Protein Coupled Estrogen Receptor Drives Functioning of Postnatal Boar Testis—Next Generation Sequencing Analysis

**DOI:** 10.3390/ani11102868

**Published:** 2021-09-30

**Authors:** Michal Duliban, Piotr Pawlicki, Artur Gurgul, Ryszard Tuz, Zbigniew Arent, Malgorzata Kotula-Balak, Kazimierz Tarasiuk

**Affiliations:** 1 Department of Endocrinology, Institute of Zoology and Biomedical Research, Jagiellonian University in Krakow, 30-387 Krakow, Poland; 2 Center of Experimental and Innovative Medicine, University of Agriculture in Krakow, 30-248 Krakow, Poland; piotr.pawlicki@urk.edu.pl (P.P.); artur.gurgul@urk.edu.pl (A.G.); 3 Department of Genetics, Animal Breeding and Ethology, Faculty of Animal Science, University of Agriculture in Krakow, 30-059 Krakow, Poland; ryszard.tuz@urk.edu.pl; 4 University Centre of Veterinary Medicine JU-UA, University of Agriculture in Krakow, 30-059 Krakow, Poland; zbigniew.arent@urk.edu.pl (Z.A.); kazimierz.tarasiuk@urk.edu.pl (K.T.)

**Keywords:** G-protein coupled estrogen receptor, peroxisome proliferator-regulated receptor, boar, testes, Next Generation Sequencing

## Abstract

**Simple Summary:**

As of now, the Next Generation Sequencing (NGS) analysis has not been utilized to identify biological processes and signaling pathways that are regulated in the boar postnatal testes. Our prior studies revealed that the peroxisome proliferator-activated receptor (PPAR) and G-protein coupled estrogen receptor (GPER) were significant for the morpho-functional status of testicular cells. Here, the pharmacological blockage of PPAR*α*, PPAR*γ* or GPER was performed in ex vivo immature boar testes. The NGS results showed 382 transcripts with an altered expression. The blockage by the PPAR*γ* antagonist markedly affected biological processes such as: drug metabolism (genes: *Ctsh*, *Duox2*, *Atp1b1*, *Acss2*, *Pkd2*, *Aldh2*, *Hbb*, *Sdhd*, *Cox3*, *Nd4*, *Nd5*, *Cytb*, *Cbr1*, and *Pid1*), adhesion (genes: *Plpp3*, *Anxa1*, *Atp1b1*, *S100a8*, *Cd93*, *Ephb4*, *Vsir*, *Cldn11*, *Gpc4*, *Fermt3*, *Dusp26*, *Sox9*, and *Cdh5*) and tube development (genes: *Ctsh*, *Mmp14*, *Dll4*, *Anxa1*, *Ephb4*, *Pkd2*, *Angptl4*, *Robo4*, *Sox9*, *Hikeshi*, *Ing2*, *Loc100738836*, and *Rarres2*), as well as the Notch signaling pathway. This was not the case for the PPAR*α* or GPER antagonists. Our observations suggested that PPARγ may be the principal player in the management of the development and function of boar testes during the early postnatal window. Moreover, due to a highly similar porcine gene expression pattern to human homologues genes, our results can be used to understand both animal and human testes physiology and to predict or treat pathological processes.

**Abstract:**

Porcine tissue gene expression is highly similar to the expression of homologous genes in humans. Based on this fact, the studies on porcine tissues can be employed to understand human physiology and to predict or treat diseases. Our prior studies clearly showed that there was a regulatory partnership of the peroxisome proliferator-activated receptor (PPAR) and the G-protein coupled membrane estrogen receptor (GPER) that relied upon the tumorigenesis of human and mouse testicular interstitial cells, as well as the PPAR-estrogen related receptor and GPER–xenoestrogen relationships which affected the functional status of immature boar testes. The main objective of this study was to identify the biological processes and signaling pathways governed by PPARα, PPARγ and GPER in the immature testes of seven-day-old boars after pharmacological receptor ligand treatment. Boar testicular tissues were cultured in an organotypic system with the respective PPARα, PPARγ or GPER antagonists. To evaluate the effect of the individual receptor deprivation in testicular tissue on global gene expression, Next Generation Sequencing was performed. Bioinformatic analysis revealed 382 transcripts with altered expression. While tissues treated with PPARα or GPER antagonists showed little significance in the enrichment analysis, the antagonists challenged with the PPARγ antagonist displayed significant alterations in biological processes such as: drug metabolism, adhesion and tubule development. Diverse disruption in the Notch signaling pathway was also observed. The findings of our study proposed that neither PPARα nor GPER, but PPARγ alone seemed to be the main player in the regulation of boar testes functioning during early the postnatal developmental window.

## 1. Introduction

Pigs provide important models for biomedical research due to sharing with humans many aspects of organ physiology, biochemistry, pathology and pharmacology. Studies by Wernersson et al. [1], demonstrated that, in pigs and humans, the number of substitutions per site separating a pair of homologous DNA sequences is very high in comparison to the common ancestral sequence in mice and humans. Hornshøj et al. [2], used 20,000 porcine transcript cDNA microarrays additionally confirmed that the gene expression pattern in porcine tissues was comparable to that of homologous human ones. The above findings justify the employment of a porcine model for comparisons with humans in transcriptomic analysis. Due to agronomical interest, pig-specific cDNA microarrays are widely available for the screening of genes involved in specific biological processes underlying the physiology and diseases of individuals [3]. It is expected that in the coming decades the pig industry will increase applied genetic selection through the determination and use of specific markers that are directly supported by effective artificial insemination technique.

The major organs of pigs and humans are the same and differ only slightly, being well-recognized even in fetuses [4]. In seminiferous tubules of postnatal testes, only gonocytes and Sertoli cells are present [5]. The abundant parenchyma with Leydig cells tightly fills the interstitial space [6]. In boars and humans, but not in other mammals, three populations of steroidogenic Leydig cells exist [7]. By 8 weeks, Leydig cells can secrete androgens into the circulation, starting the masculinization programming window that includes: development of the anogenital distance, external genitalia, urethral structures, testes descent and adult fertility [8]. However, boar postnatal testes physiology is still not investigated to the full extent.

The ligand-inducible transcription factors, peroxisome proliferator-activated receptors (PPARs), are members of the nuclear receptor superfamily. Three PPAR subtypes are identified: PPARα (*NR1C1*), PPARβ/δ (*NR1C2*) and PPARγ (*NR1C3*) [9]. The receptor of the α type was identified in 1990 in mouse and named by its ability to become activated by chemicals involving peroxisome proliferation [10]. The two other PPAR subtypes, PPARβ/δ and PPARγ were recognized by homology screens [11]. Of note, peroxisomes are oxidative organelles engaged in lipid metabolism and the conversion of reactive oxygen species [12]. While PPARα- and PPARβ/γ-null mice are viable and fertile. Therefore, PPARs are widely studied as connectors of energy metabolism and reproduction [13]. Over the past decade, numerous in vivo and in vitro studies have strongly advocated that these nuclear receptors might be of significance in the gametogenesis, parturition, gestation and interaction within the mother–fetus unit [14]. Within cells of the male reproductive system, PPARs are broadly distributed [6,15]. Indeed, in the testes, the β-oxidation of fatty acids is important for, e.g., sex steroid synthesis or spermatozoon lipid membrane composition modifications. It was demonstrated that in human spermatozoa, PPARγ was implicated in the motility and acrosome reaction [16]. In Sertoli cells, PPARα and PPAR β/δ are required for cell metabolism and estrogen production [17,18]. It was confirmed, that either sex hormones or some endocrine-disrupting chemicals, such as phthalates, acted via PPARα and PPARγ, leading to the modulation of PPAR activities [19]. In mouse Leydig cells, Gazouli et al. [20] discovered the contribution of cholesterol-transporting proteins in the regulation of steroidogenesis by PPARα. Moreover, Kowalewski et al. [21] demonstrated that the activation of PPARγ by its ligand stopped steroidogenesis in these cells. Our recent studies showed changes in the number of lipid droplets and the ultrastructure of mitochondria in PPARγ-blocked immature boar Leydig cells [6].

It is worth mentioning that a new generation of PPAR-targeting pharmacological drugs is undergoing clinical trials for the treatment of infertility associated with metabolic disorders such as insulin resistance [14]. In addition, PPAR ligands are recommended to be used for the amelioration of preeclampsia during hypertension and inflammation. Moreover, the success rate of the in vitro fertilization method might be enhanced in the coming years by the enrichment of the culture media with PPARβ/δ ligands [22]. In prostate and testicular cancers, PPARγ constitutes a potent target to treat and prevent these diseases [23].

The membrane G-protein coupled estrogen receptor (GPER) is the first sensor for the endogenous and environmental action of estrogenic compounds, e.g., BPA (bisphenol A) that triggers different intracellular targets through the activation of fast nongenomic mechanisms [24]. Several recent studies revealed GPER presence and its implication in the testes functions of various animals [25,26,27,28]. Moreover, the association of GPER and estrogen-related receptors or PPAR interaction in the control of healthy and tumor testicular cells in rodents and humans was previously demonstrated by us [6,29,30,31]. Nowadays, the GPER antagonist is monitored as a potential therapeutic drug for male infertility treatment [32]. Nevertheless, the mechanisms of both PPAR and GPER action remain unclear and the potential long-term adverse effects in fertility control are unknown. Therefore, further investigations are urgently needed.

The present research was focused on the determination of genes and gene-controlled molecular processes in the immature boar testes after PPARα, PPARγ or GPER pharmacological antagonist treatment. The global gene expression analysis of differentially regulated transcripts was followed by a cluster analysis and a pathway analysis, respectively. This included an analysis of differentially regulated genes and proteins expressed in testes with a known morphological status that were partially earlier studied by us [6,28,29,30,31,33]. To the best of the authors’ knowledge, no research has engaged in this type of approach before.

## 2. Materials and Methods

### 2.1. Tissue Collection and Ex Vivo Culture

In the Republic of Poland, male piglets are routinely castrated in the early days after birth to avoid the production of the main testicular androgen, androstenone, which accumulates in fat and results in the development of the boar taint. The testes (*n* = 40) of 7-day-old Polish White Large boars (*n* = 20) from a breeding farm in Malopolska, Krakow, Poland were obtained during anaesthetized surgical castration of animals. Tissues were transported to the laboratory in Dulbecco’s phosphate-buffered saline (DPBS, Sigma-Aldrich, St Louis, MO, USA) supplemented with 2% penicillin-streptomycin solution (Invitrogen, Carlsbad, CA, USA) within 1 h. From the preautoclaved 1.5% agarose, small pillars were prepared a day before the experiment. After solidification agarose was cut into columns (approx. 8 mm width and 5 mm height), the columns were immersed in the Dulbecco’s modified Eagle medium (DMEM; Sigma-Aldrich; St Louis, MO, USA). Three column per well were placed into the six-well plates. Small testicular pieces (approx. 2 mm) were located on top of the pillars (one piece per pillar) in DMEM supplemented with 10% fetal bovine serum (FBS) (Sigma-Aldrich; St Louis, MO, USA) and L-glutamine, 50 U/mL penicillin, and 50 μg/mL streptomycin (without phenol red and with the addition of 5% dextran-coated, charcoal-treated FBS to exclude estrogenic effects caused by the medium). Testicular explants were incubated at 32 °C (to protect seminiferous tubule epithelium) in an atmosphere containing 95% air: 5% CO_2_. Selective PPARα antagonist (N-((2S)-2-(((1Z)-1-Methyl-3-oxo-3-(4-(trifluoromethyl) phenyl)prop-1-enyl)amino)-3-(4-(2-(5-methyl-2-phenyl-1,3-oxazol-4-yl)ethoxy)phenyl)propyl) propanamide, GW6471) (Tocris Bioscience, Bristol, UK), PPARγ antagonist (2-Chloro-5-nitro-N-4-pyridinyl benzamide, T0070907) (Sigma-Aldrich, Missouri, MO, USA) or GPER antagonist ((3aS*,4R*,9bR*)-4-(6-Bromo-1,3-benzodioxol-5-yl)-3a,4,5,9b-3H-cyclopenta(c)quinolone; G15) (Tocris Bioscience, Bristol, UK) were dissolved in dimethyl sulfate (DMSO). Stock solutions were shortly stored at −20 °C. Concentration of chemicals used for tissue treatment was determined during preliminary experiments and previous studies (for details see [29,31,33]). The DMSO concentration within the culture medium was <0.1% (*v*/*v*). Control tissues were incubated with medium including only the solvent. Pieces of testicular tissues in separate wells of culture plate were treated with respective antagonist [PPARα (10 μM) or PPARγ (10 μM) or G15 (10 nM)] for 24 h. Experiments were performed three times, each in triplicate.

The use of boar testes after surgical castration (according to European Union Council Directive 2010-63-EU) was approved by the Local Ethics Committee in Krakow, Poland (permission number: 144b/2015).

After ex vivo experiment boar testicular tissues (*n* = *12*) were immediately frozen and stored in −80 °C. Samples were homogenized in 1 mL TRIzol chemical (Invitrogen; Carlsbad, CA, USA). The isolation and purification of RNA were performed using a RNeasy Mini Kit (Qiagen; Germantown, MD, USA) accordingly to the manufacturer’s manual. The total RNA concentration was measured using a ND-100 Spectrometer (NanoDrop Technologies, Wilmington, DE, USA). The quality of RNA was estimated using an Agilent Bioanalyzer 2100(Agilent Technologies, Santa Clara, CA, USA). It did not raise any concerns (RIN > 8.0).

### 2.2. Library Preparation and NGS

Sequencing of RNA (RNA-seq) was conducted commercially by Intelliseq Biotechnological Company (Krakow, Poland). For mRNA sequencing, libraries were generated using an Illumina TruSeq Stranded mRNA Library Prep Kit. cDNA libraries were sequenced using a HiSeq4000 (Illumina, San Diego, CA, USA) with the following parameters: PE150 (150-bp paired end) and a minimum of 40 million (40 M) raw reads.

### 2.3. Data Analysis

For the evaluation of raw sequencing reads, quality FastQ software (Babraham Bioinformatics, Cambridge, UK) was used. Obtained reads displayed acceptable quality and no overrepresentation of adaptor sequences was detected. Subsequently the reads were mapped against the Ensembl Sscrofa11.1 genome built with Hisat2 2.1.0 software (Baltimore, MD, USA http://daehwankimlab.github.io/hisat2/ accessed on 20 February 2020) [34]. For estimation of transcripts abundance, Cuffquant and Cuffmerge v.2.2.1 (http://cole-trapnell-lab.github.io/cufflinks/install/) [35] software was used along with the GTF annotation file (Sus_scrofa.Sscrofa11.1.98.gtf) from the Ensembl database. For normalization and calculation of fragments per kilobase of exon per million reads mapped (FPKM), Cuffmerge (Trapnell Lab, Seattle, WA, USA) software with the library-norm-method classic-fpkm option was run. Prior to the analysis of genes with differential expression, data were filtered to remove transcripts where the expression level was not measured (FPKM = 0). For further analysis, 51,217 transcripts were used. To detect the genes with a different expression between the experimental groups, one-way ANOVA test was performed. The obtained P-values were corrected for multiple testing by employing a false discovery rate (FDR) method [36]. Expression profiles of the samples were compared and clustered. The principal components analysis (PCA) and unsupervised hierarchical clustering were prepared based on Euclidean distance using ClustVis online software (http://biit.cs.ut.ee/clustvis/ accessed on 15 June 2021) [37].

All transcripts with differential expressions were analyzed regarding: their molecular functions, cellular components, associated biological processes, and KEGG pathways, with the use of the web-based GEne SeT AnaLysis Toolkit (http://www.webgestalt.org/ accessed on 20 June 2021) [38]. Enrichment of gene set was analyzed based on all known *Sus scrofa* transcripts with FDR correction for multiple testing.

For all analyzed samples, the raw sequencing reads are available through the SRA (Sequence Read Archive) NCBI database under the accession number PRJNA750794.

## 3. Results

### 3.1. Mapped Reads, Statistics and Global Expression Profiles

**Figure 1:**
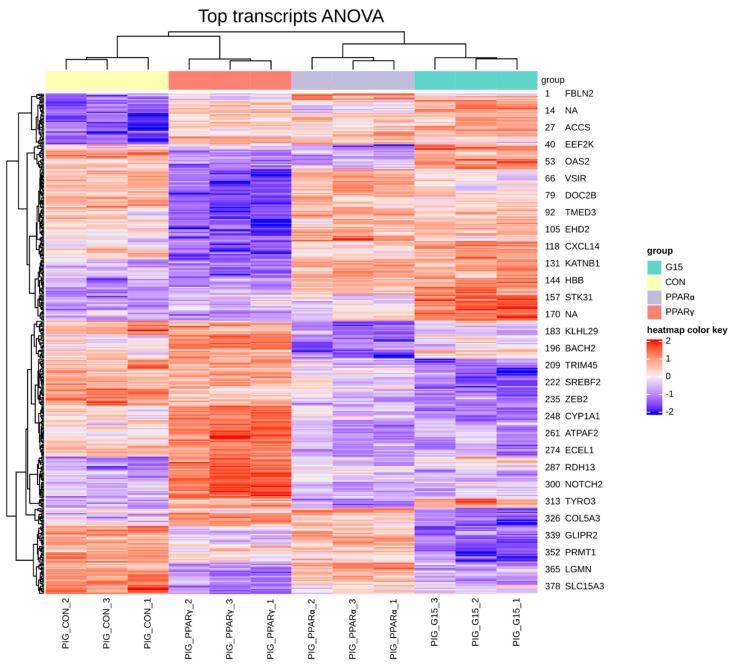
Hierarchical clustering and heatmap of expression profiles for samples and genes (383) with altered expression. Global ANOVA test across PPARα, PPARγ and GPER antagonist-treated groups. (PIG_CON—control group, PIG_PPARα– group treated with PPARα antagonist, PIG_PPARγ—group treated with PPARγ antagonist, PIG_G15group treated with G15 antagonist).

Between 40.0 × 10^6^ and 49.0 × 10^6^ of raw paired-end reads per sample were generated during sequencing. In all analyzed samples, the mean mapping efficiency was satisfactory and exceeded 76. The FPKM normalization and transcript filtering retained 51 and 217 transcripts for further analysis. The differences in the expression profiles between all PPARα PPARγ, and GPER antagonist-treated groups revealed 383 transcripts that differed significantly (FDR < 0.1). Using hierarchical clustering, the sample expression profiles were clearly allocated into separate groups suggesting a distinct pattern of changes in transcript expression between the analyzed groups (Figure 1).

**Figure 2:**
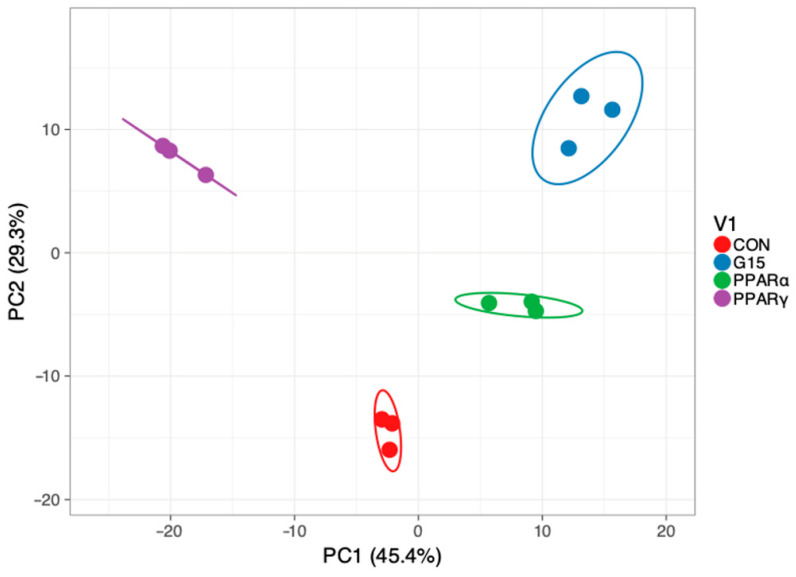
Principal component analysis and hierarchical clustering of samples based on genes with altered expressions. Global ANOVA test across PPAR*α*, PPAR*γ* and GPER antagonist-treated groups. (CON—control group, PPAR*α*—group treated with PPAR*α* antagonist, PPAR*γ*—group treated with PPAR*γ* antagonist, G15group treated with G15 antagonist).

Based on the principal component analysis of genes expression profiles, a sharp separation of the studied groups was revealed (Figure 2).

From 383 transcripts, 378 transcripts belonged to unique genes. Prior enrichment analysis of the biological processes revealed that 360 transcripts were unambiguously mapped to entrezgene IDs. The differentially expressed genes significantly enriched biological processes such as: drug metabolism (e.g., genes: *Ctsh*, *Duox2*, *Atp1b1*, *Acss2*, *Pkd2*, *Aldh2*, and *Cox1*), oxidation–reduction (e.g., genes: *Cyp21a2*, *Nqo1*, *Duox2*, *Sqle*, *Lox*, and *Cox3*) and negative regulation of signaling (e.g., genes: *Ybx3*, *Ripk1*, *Dll4*, *Ephb4*, *Taok3*, *Sox9*, and *Ing2*).

### 3.2. Effect of PPARγ Antagonist on Gene Expression

**Figure 3:**
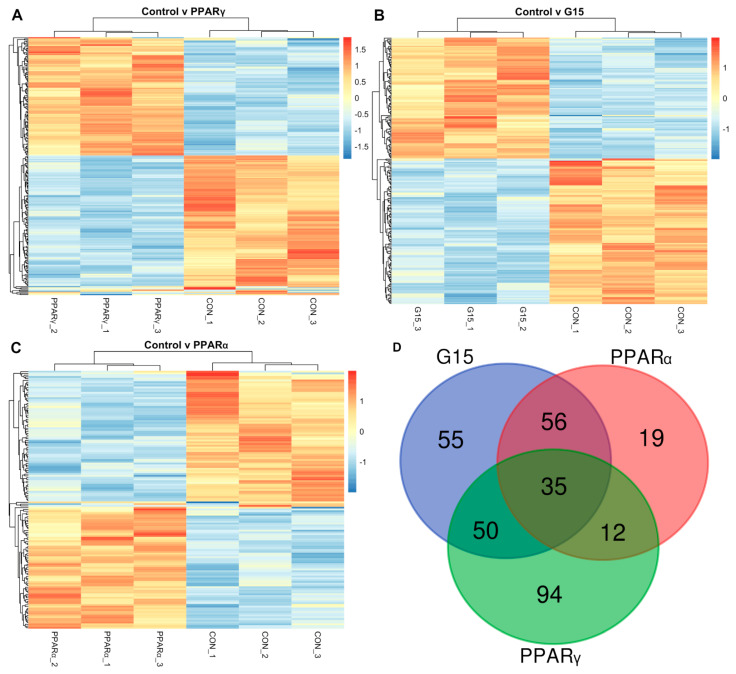
Expression profiles based on genes differentially expressed between control group (CON) and and (**A**) group treated with PPAR*γ* antagonist (PPAR*γ*), (**B**) G15group treated with G15 antagonist (G15) and (**C**) group treated with PPAR*α* antagonist (PPAR*α*). See Results (paragraph 3.5) for differentially expressed genes. (**D**)Venn diagram demonstrating the number of differentially expressed genes in experimental groups (PPARα—group treated with PPARα antagonist, PPARγ—group treated with PPARγ antagonist, G15—group treated with G15 antagonist).

The treatment of boar testes with the PPARγ antagonist caused changes in the expressions of 229 transcripts belonging to 226 different genes when compared to control (Figure 3A).

The majority of the genes in the PPARγ experimental group were downregulated (*n* = 122; 53.9%). The genes enriched significantly biological processes corresponding to: e.g., drug metabolism (*Ctsh*, *Duox2*, *Atp1b1*, *Acss2*, *Pkd2*, *Aldh2*, *Hbb*, *Sdhd*, *Cox3*, *Nd4*, *Nd5*, *Cytb*, *Cbr1*, and *Pid1*), biological adhesion (*Plpp3*, *Anxa1*, *Atp1b1*, *S100a8*, *Cd93*, *Ephb4*, *Vsir*, *Cldn11*, *Gpc4*, *Fermt3*, *Dusp26*, *Sox9*, and *Cdh5*) and tube development (*Ctsh*, *Mmp14*, *Dll4*, *Anxa1*, *Ephb4*, *Pkd2*, *Angptl4*, *Robo4*, *Sox9*, *Hikeshi*, *Ing2*, *Loc100738836*, and *Rarres2*). The genes were mainly overrepresented in cellular components related to respiratory chain complex (FDR < 0.05) and extracellular organelle (Supplementary File S1). The separate functional analysis of the 104 up- and 122 downregulated genes revealed that the upregulated genes were mainly engaged in biological processes responsible for the regulation of transporter activity. However, they were also enriched in response to toxic substances, while downregulated genes were responsible mainly for drug metabolism, tube development and locomotion (FDR < 0.1). The analysis of gene set involvement in biological pathways revealed that differentially expressed genes significantly (FDR < 0.1) enriched cellular metabolic processes such as: Histidine metabolism, β-Alanine metabolism, Pyruvate metabolism, and Glycolysis/Gluconeogenesis. Additionally, we identified a disruption in the Notch signaling pathway with an FDR = 0.13 (trend). This will be furtherly explored in the Discussion section.

### 3.3. The Effect of GPER Antagonist on Gene Expression

The administration of the GPER antagonist (G15) resulted in the altered expressions (with respect to the control) of 225 transcripts. They belonged to different genes (Figure 3B).

The genes did not enrich statistically (after multiple testing correction, FDR > 0.6) for any molecular functions, biological processes, or cellular components; however, they showed pointwise enrichments of these processes, such as: drug metabolism, and vasculature development (e.g., *Eng*, *Anxa1*, *Itga5*, *Sphk1*, and *Ctsh*). Cellular components with pointwise enrichments mainly represented the cell surface and extracellular organelle (Supplementary File S1). The molecular functions of the genes represented protein binding, ion binding, nucleic acid binding and transferase activity. The separate analysis of up- and downregulated genes revealed 124 upregulated genes that enriched (only at pointwise level) the biological processes related to the regulation of protein stability (*Ctsh*, *Plpp3*, *Rassf2*, *Casp3*, and *Bmp2*) and tissue remodeling (*Dll4*, *Anxa1*, *Loc100738836*, and *Rassf2*). The downregulated 101 genes mainly included processes responsible for cofactor metabolism or purine-containing compound metabolism, and for enriched cellular components being an essential component of the synaptic membrane or cytoskeleton-associated proteins. The KEGG pathway analysis of gene sets did not reveal any significant pathways but showed a similar trend of the pointwise significance in metabolic pathways.

### 3.4. The Effect of PPARα Antagonist on Gene Expression

In testes treated with the PPARα antagonist, 146 transcripts showed different expressions (FDR < 0.1). These transcripts belonged to 143 different genes (Figure 3C).

Of the transcripts, 72 were upregulated and 74 were downregulated with reference to the control. The common function analysis of up- and downregulated genes displayed that only at the pointwise level did the genes enrich biological processes involved in drug metabolism and the response to an abiotic stimulus. The subset of genes upregulated by the blockage of PPARα-enriched biological processes such as: the regulation of the oxidoreductase activity and cellular macromolecule localization; however, it did not withstand an FDR correction (FDR = 1). The downregulated genes did not show significant associations after FDR correction; however, they presented the same trend in the enrichment of biological processes as the whole gene set (Supplementary File S1).

### 3.5. Comparative Analysis of Genes after Treatment with PPARα, PPARγ and GPER Antagonist

The comparative analysis of genes with differential expressions in PPARα, PPARγ and GPER and antagonist-treated groups revealed that 35 out of 220 genes (affected in total) were altered (Figure 3D).

These genes showed no strong and visible trend in the enrichment of cellular components and were not significantly overrepresented in any molecular functions or biological processes. The genes that were altered solely by both the administrations of GPER or PPARα antagonists comprised 56 different entries. These genes displayed no significant tendency (FDR = 1) to the enrichment of any biological process, cellular components or molecular function. The genes that were altered by both the PPARγ antagonist or the PPARα antagonist (*n* = 12) showed a strong overrepresentation (FDR < 0.01) in biological processes affecting metanephros morphogenesis (Pkd2, Sox9) such as development of the metanephric tubule, metanephric epithelium and metanephric nephron. However, these genes did not significantly enrich any cellular components or molecular functions.

## 4. Discussion

In the present study, only a cell-permeable, chloro-nitro-benzamido compound with potent, specific, irreversible, and high-affinity antagonistic properties to PPARγ affected a significant number of genes involved in the important biological pathways in immature boar testes. The results obtained from testes treated with PPARα or GPER antagonists showed a little to non-statistical significance after the functional enrichment of the gene lists. This might suggest that, at this developmental stage of boar testes, PPARα and GPER are of a lesser importance for the postnatal testes functioning. Another possible explanation may involve the mapping efficiency and porcine genome description. While the average mapping efficiency for the mouse or human genome is usually 80–90%, in the case of this experiment, 75% is relatively lower than in other experiments. This might be caused by the paired-end approach and/or the usage of the stranded kit for which the mapping efficiency is slightly lower [39,40]. Additionally, it is worth noting the importance of the genome description. From 382 transcripts, 56 transcripts still do not have an assigned gene name and thus cannot be used in functional enrichment. The unusual ligand-binding properties of PPARγ are well-known and used in the treatment of type 2 diabetes and other metabolic disorders [41]. The present results show that the PPARγ antagonist is actively metabolized by the testicular cells of an immature boar. From a functional perspective, the blockage of a receptor can cause similar, but temporary, effects such as gene knockout. The complete knockdown of Pparγ is lethal [42] and can cause changes in perigonadal fat deposition and insulin resistance in mice [43]. Recent studies demonstrated that PPARγ was important in glucose utilization [44]. Similarly, in the analysis of gene engagement and the involvement of PPARγ in pathways, Glycolysis/Gluconeogenesis (Supplementary Material S1) was observed. Additionally, we distinguished an expression change of a number of genes involved in metabolic processes: amino acids metabolism (histidine, β-alanine) and vitamin digestion and absorption. This further confirms a crucial role of PPARγ in cell metabolism [45].

Here, the pharmacological deprivation of PPARγ affects the adhesion and migration properties of testicular cells that are crucial for proper spermatogenesis. The detected disruption in the expression of *Fermt3* may affect the adhesive properties of cells. The genetic alterations in *Fermt3* were found to alter the adherent properties of integrin [46]. There is evidence showing that the genetic mutations in *Fermt3* lead to changes in integrin activation that can further cause leukocyte adhesion deficiency [47,48]. Claudins cover a large family of the tight junction protein. *Cldn11* is known to be vital for normal spermatogenesis [49]. In mice overexpressing *Cldn11*, the functions of Sertoli cells were not disturbed, and no gaining of morphological phenotypes was observed [50]. In immature testes treated with PPARγ, we also revealed an overexpression of *Cldn11*. Other findings showed that this alteration did not change the phenotype of cells [50]. The processes would be strongly active and would be regulated at this stage of boar testes development under PPARγ supervision. In the testes, like in other tissues, cell adhesion was achieved via cell junctions composed of adhesion molecules eliciting the appropriate changes in cell adhesion in response to environmental stimuli [51]. Without cell adhesion, the sloughing of spermatogenic cells into seminiferous tubule lumen occurs and results in serious fertility problems. In human vascular endothelial cells, the constitutive activation of PPARγ suppresses pro-inflammatory adhesion molecules [52]. Shen et al. [53] reported that PPARγ inhibits hepatocellular carcinoma metastases in vitro in mice through the upregulation of adhesion molecules: E-cadherin and spleen tyrosine kinase. In mouse tumor Leydig cells, we previously demonstrated the GPER-PPARα partnership through the PI3K/Akt pathway, and the effect of the GPER-PPARγ via the Ras/Raf pathway on the cytoskeleton structure, migration competences and morphology of these cells [29]. In rheumatoid arthritis, GPER was also involved in the proliferation and migration of fibroblast-like synoviocytes [54]. Similarly, Goetze et al. [55] found that PPARγ ligands inhibited vascular smooth muscle cell migration mediated by multiple chemoattractants. In human testicular cancer, PPARγ is induced by its ligands mediating potent antiproliferative effects through differentiation [56].

In immature boar testes, PPARγ governs further seminiferous tubule development. Indeed, a number of developmental events, both structural and molecular, take place in the testes throughout the second and third postnatal weeks, e.g., the development of peritubular-myoid cells; onset of the first wave of meiosis; maturation of Sertoli cells, including the formation of their specialized junctions of the blood–testes barrier; canalization of seminiferous cords; and increased Sertoli cell secretion [57]. Early findings by Kosco et al. [58] demonstrated that, in neonatal hemicastrated boars, due to Sertoli cell proliferation, an earlier onset of spermatogenesis, rapid, compensatory and seminiferous tubule elongation occurred. However, gonocytes proliferated only after they transform into spermatogonia. In human and rat testes, PPARα mRNA and protein expression increased toward adulthood in both seminiferous tubule cells and Leydig cells [15]. Our findings implied that PPARα could be partially involved in the differentiation and growth regulation of tubular and interstitial cells, such as in rat and human testes [13]. Rosiglitazone treatment attenuated tubulointerstitial fibrosis and the epithelial phenotype transition in wild type mice but not diminished proximal tubule of PPARγ knockout mice [59]. These findings identified an important role of renal tubular epithelium-targeted PPARγ in maintaining the normal epithelial phenotype and opposing fibrogenesis via antagonizing oxidative stress.

In this study we identified disruptions in the expression of four genes (*Notch2*, *Maml3*, *Notch1*, and *Dll4*) involved in the Notch signaling pathway in testicular tissue with blocked PPARγ. Interestingly, the expression of *Notch2* and *Maml3* was elevated, and *Notch1* and *Dll4* expression decreased. *Maml3* (*Mastermind-like 3*) is a conserved nuclear factor that was demonstrated as necessary for Notch signaling *in vivo*, but the loss of *Maml3* caused no visible defects in mice [60]. The alterations in the expression pattern of the components of the Notch pathway and the replacement of Notch1 receptor by Notch3 were detected in Sertoli cells throughout the postnatal development of mouse testes [61]. To date, the meaning and regulation of Notch2 was demonstrated in fetal mouse testes and in tumor Leydig cells [62]. In addition, it was previously reported that testosterone directly regulated Notch signaling in progenitor Leydig cells and sustained the fetal Leydig cell population [63]. Although, the function of androgens in the control of the Notch pathway in seminiferous epithelium was partially explored [64]. Another report implicates the involvement of Notch signaling in the interactions of 17 β-estradiol and angiogenesis in breast cancer cells [65]. Male infertility was associated with aberrant Notch activity in rodents and humans [66]. Surprisingly, Hasegawa et al. [67] reported that in mouse testes, most of Notch pathway components were not transcribed, and thus the Notch blockage in germ and Sertoli cells did not impact spermatogenesis.

## 5. Conclusions

Our study, for the first time, provides transcriptomic insights into PPAR and GPER roles in postnatal boar testes physiology. The applied bioinformatic analysis revealed 382 transcripts with altered expressions after treatment with respective receptor antagonists. In general, ex vivo testicular tissues treated with the PPARγ antagonist displayed significant alterations in the processes of drug metabolism, biological adhesion, and tubule development, as well as a diverse disruption of the Notch signaling pathway. Therefore, it is suggested that PPARγ may be the main player, while the roles of PPARα and GPER are most likely secondary in the regulation of the early developmental window of boar testes. The novel and interesting in silico results that we obtained warrant further research across the whole field of experimental andrology.

The main limitation of the study is the number of samples used for the Next Generation Sequencing es analysis. This is due to the high costs of this analysis limited by the grant project funds. In addition, the validation of the results by qRT-PCR at the protein level are important future research tasks. The employment of an extended epigenetic analysis might provide explanations regarding gene expression changes.

## Data Availability

Data available in a publicly accessible repository that does not issue DOIs. The raw sequencing reads are available through the SRA (Sequence Read Archive) NCBI database under the accession number PRJNA750794.

## Supplementary Materials

The following are available online at https://www.mdpi.com/article/10.3390/ani11102868/s1, File S1: Genes differentially expressed across study groups as showed by ANOVA.

## References

[1] Wernersson R., Schierup M.H., Jørgensen F.G., Gorodkin J., Panitz F., Staerfeldt H.H., Christensen O.F., Mailund T., Hornshøj H., Klein A. (2005). Pigs in sequence space: A 0.66X coverage pig genome survey based on shotgun sequencing. BMC Genom..

[2] Hornshøj H., Conley L.N., Hedegaard J., Sørensen P., Panitz F., Bendixen C. (2007). Microarray expression profiles of 20.000 genes across 23 healthy porcine tissues. PLoS ONE.

[3] Moe M., Meuwissen T., Lien S., Bendixen C., Wang X., Conley L.N., Berget I., Tajet H., Grindflek E. (2007). Gene expression profiles in testis of pigs with extreme high and low levels of androstenone. BMC Genom..

[4] O’Shaughnessy P.J., Fowler P.A. (2014). Development of the human fetal testis. Ann. Endocrinol..

[5] Pontelo T.P., Miranda J.R., Felix M.A.R., Pereira B.A., da Silva W.E., Avelar G.F., Mariano F.C.M.Q., Guimarães G.C., Zangeronimo M.G. (2018). Histological characteristics of the gonads of pig fetuses and their relationship with fetal anatomical measurements. Res. Vet. Sci..

[6] Kotula-Balak M., Gorowska-Wojtowicz E., Milon A., Pawlicki P., Tworzydlo W., Płachno B.J., Krakowska I., Hejmej A., Wolski J.K., Bilinska B. (2020). Towards understanding leydigioma: Do G protein-coupled estrogen receptor and peroxisome proliferator-activated receptor regulate lipid metabolism and steroidogenesis in Leydig cell tumors?. Protoplasma.

[7] Raeside J.I., Renaud R.L. (1983). Estrogen and androgen production by purified Leydig cells of mature boars. Biol. Reprod..

[8] Dean A., Sharpe R.M. (2013). Clinical review: Anogenital distance or digit length ratio as measures of fetal androgen exposure: Relationship to male reproductive development and its disorders. J. Clin. Endocrinol. Metab..

[9] Varga T., Czimmerer Z., Nagy L. (2011). PPARs are a unique set of fatty acid regulated transcription factors controlling both lipid metabolism and inflammation. Biochim. Biophys. Acta.

[10] Tyagi S., Gupta P., Saini A.S., Kaushal C., Sharma S. (2011). The peroxisome proliferator-activated receptor: A family of nuclear receptors role in various diseases. J. Adv. Pharm. Technol. Res..

[11] Bugge A., Mandrup S. (2010). Molecular mechanisms and genome-wide aspects of PPAR subtype specific transactivation. PPAR Res..

[12] Poirier Y., Antonenkov V.D., Glumoff T., Hiltunen J.K. (2006). Peroxisomal beta-oxidation—A metabolic pathway with multiple functions. Biochim. Biophys. Acta.

[13] Froment P., Gizard F., Defever D., Staels B., Dupont J., Monget P. (2006). Peroxisome proliferator-activated receptors in reproductive tissues: From gametogenesis to parturition. J. Endocrinol..

[14] Froment P. (2008). PPARs and RXRs in male and female fertility and reproduction. PPAR Res..

[15] Schultz R., Yan W., Toppari J., Völkl A., Gustafsson J.A., Pelto-Huikko M. (1999). Expression of peroxisome proliferator-activated receptor alpha messenger ribonucleic acid and protein in human and rat testis. Endocrinology.

[16] Liu L.L., Xian H., Cao J.C., Zhang C., Zhang Y.H., Chen M.M., Qian Y., Jiang M. (2015). Peroxisome proliferator-activated receptor gamma signaling in human sperm physiology. Asian J. Androl..

[17] Regueira M., Riera M.F., Galardo M.N., Pellizzari E.H., Cigorraga S.B., Meroni S.B. (2014). Activation of PPAR α and PPAR β/δ regulates Sertoli cell metabolism. Mol. Cell Endocrinol..

[18] Hassanpour H., Khalaji-Pirbalouty V., Adibi M., Nazari H. (2017). Involvement of peroxisome proliferator-activated receptors in the estradiol production of ovine Sertoli cells. Vet. Res. Forum.

[19] Latini G., Scoditti E., Verrotti A., de Felice C., Massaro M. (2008). Peroxisome proliferator-activated receptors as mediators of phthalate-induced effects in the male and female reproductive tract: Epidemiological and experimental evidence. PPAR Res..

[20] Gazouli M., Yao Z.X., Boujrad N., Corton J.C., Culty M., Papadopoulos V. (2002). Effect of peroxisome proliferators on Leydig cell peripheral-type benzodiazepine receptor gene expression, hormone-stimulated cholesterol transport, and steroidogenesis: Role of the peroxisome proliferator-activator receptor alpha. Endocrinology.

[21] Kowalewski M.P., Dyson M.T., Manna P.R., Stocco D.M. (2009). Involvement of peroxisome proliferator-activated receptor gamma in gonadal steroidogenesis and steroidogenic acute regulatory protein expression. Reprod. Fertil. Dev..

[22] Huang J.C., Wun W.S., Goldsby J.S., Wun I.C., Noorhasan D., Wu K.K. (2007). Stimulation of embryo hatching and implantation by prostacyclin and peroxisome proliferator-activated receptor delta activation: Implication in IVF. Hum. Reprod..

[23] Matsuyama M., Yoshimura R. (2008). Peroxisome Proliferator-Activated Receptor-gamma Is a Potent Target for Prevention and Treatment in Human Prostate and Testicular Cancer. PPAR Res..

[24] Vögler O., Barceló J.M., Ribas C., Escribá P.V. (2008). Membrane interactions of G proteins and other related proteins. Biochim. Biophys. Acta.

[25] Chimento A., Sirianni R., Casaburi I., Pezzi V. (2014). Role of estrogen receptors and g protein-coupled estrogen receptor in regulation of hypothalamus-pituitary-testis axis and spermatogenesis. Front. Endocrinol..

[26] Vaucher L., Funaro M.G., Mehta A., Mielnik A., Bolyakov A., Prossnitz E.R., Schlegel P.N., Paduch D.A. (2014). Activation of GPER-1 estradiol receptor downregulatesproduction of testosterone in isolatedrat Leydig cells and adult human testis. PLoS ONE.

[27] Zarzycka M., Gorowska-Wojtowicz E., Tworzydlo W., Klak A., Kozub K., Hejme A., Bilinska B., Kotula-Balak M. (2016). Are aryl hydrocarbon receptor and G-protein-coupled receptor 30 involved in the regulation of seasonal testis activity in photo-sensitive rodent-the bank vole (Myodes glareolus)?. Theriogenology.

[28] Kotula-Balak M., Pawlicki P., Milon A., Tworzydlo W., Sekula M., Pacwa A., Gorowska-Wojtowicz E., Bilinska B., Pawlicka B., Wiater J. (2018). The role of G-protein-coupled membrane estrogen receptor in mouse Leydig cell function-in vivo and in vitro evaluation. Cell Tissue Res..

[29] Gorowska-Wojtowicz E., Dutka P., Kudrycka M., Pawlicki P., Milon A., Plachno B.J., Tworzydlo W., Pardyak L., Kaminska A., Hejmej A. (2018). Regulation of steroidogenic function of mouse Leydig cells: G-coupled membrane estrogen receptor and peroxisome proliferator-activated receptor partnership. J. Physiol. Pharmacol..

[30] Pawlicki P., Duliban M., Tuz R., Ptak A., Milon A., Gorowska-Wojtowicz E., Tworzydlo W., Płachno B.J., Bilinska B., Knapczyk-Stwora K. (2019). Do G-protein coupled estrogen receptor and bisphenol A analogs influence on Leydig cell epigenetic regulation in immature boar testis ex vivo?. Anim. Reprod. Sci..

[31] Kotula-Balak M., Duliban M., Pawlicki P., Tuz R., Bilinska B., Plachno B.J., Arent Z.J., Krakowska I., Tarasiuk K. (2020). The meaning of non-classical estrogen receptors and peroxisome proliferator-activated receptor for boar Leydig cell of immature testis. Acta Histochem..

[32] Zhang D., Wang Y., Lin H., Sun Y., Wang M., Jia Y., Yu X., Jiang H., Xu W., Sun J.P. (2020). Function and therapeutic potential of G protein-coupled receptors in epididymis. Br. J. Pharmacol..

[33] Pawlicki P., Hejmej A., Milon A., Lustofin K., Płachno B.J., Tworzydlo W., Gorowska-Wojtowicz E., Pawlicka B., Kotula-Balak M., Bilinska B. (2019). Telocytes in the mouse testicular interstitium: Implications of G-protein-coupled estrogen receptor (GPER) and estrogen-related receptor (ERR) in the regulation of mouse testicular interstitial cells. Protoplasma.

[34] Pertea M., Kim D., Pertea G.M., Leek J.T., Salzberg S.L. (2016). Transcript-level expression analysis of RNA-seq experiments with HISAT, StringTie and Ballgown. Nat. Protoc..

[35] Trapnell C., Williams B.A., Pertea G., Mortazavi A., Kwan G., van Baren M.J., Salzberg S.L., Wold B.J., Pachter L. (2010). Transcript assembly and quantification by RNA-Seq reveals unannotated transcripts and isoform switching during cell differentiation. Nat. Biotechnol..

[36] Benjamini Y., Hochberg Y. (1995). Controlling the false discovery rate: A practical and powerful approach to multiple testing. J. R. Stat. Soc. Ser. B.

[37] Metsalu T., Vilo J. (2015). ClustVis: A web tool for visualizing clustering of multivariate data using Principal Component Analysis and heatmap. Nucleic Acids Res..

[38] Liao Y., Wang J., Jaehnig E.J., Shi Z., Zhang B. (2019). WebGestalt 2019: Gene set analysis toolkit with revamped UIs and APIs. Nucleic Acids Res..

[39] Zhao S., Zhang Y., Gordon W., Quan J., Xi H., Du S., von Schack D., Zhang B. (2015). Comparison of stranded and non-stranded RNA-seq transcriptome profiling and investigation of gene overlap. BMC Genom..

[40] Oczkowicz M., Świątkiewicz M., Ropka-Molik K., Gurgul A., Żukowski K. (2016). Effects of Different Sources of Fat in the Diet of Pigs on the Liver Transcriptome Estimated by RNA-Seq. Ann. Anim. Sci..

[41] Willson T.M., Lambert M.H., Kliewer S.A. (2001). Peroxisome proliferator-activated receptor gamma and metabolic disease. Annu. Rev. Biochem..

[42] Barak Y., Nelson M.C., Ong E.S., Jones Y.Z., Ruiz-Lozano P., Chien K.R., Koder A., Evans R.M. (1999). PPAR gamma is required for placental, cardiac, and adipose tissue development. Mol. Cell..

[43] Tsai Y.S., Tsai P.J., Jiang M.J., Chou T.Y., Pendse A., Kim H.S., Maeda N. (2009). Decreased PPAR gamma expression compromises perigonadal-specific fat deposition and insulin sensitivity. Mol. Endocrinol..

[44] Karak M., Bal N.C., Bal C., Sharon A. (2013). Targeting peroxisome proliferator-activated receptor gamma for generation of antidiabetic drug. Curr. Diabetes Rev..

[45] Gray S.L., dalla Nora E., Vidal-Puig A.J. (2005). Mouse models of PPAR-gamma deficiency: Dissecting PPAR-gamma’s role in metabolic homoeostasis. Biochem. Soc. Trans..

[46] Zimmerman G.A. (2009). LAD syndromes: FERMT3 kindles the signal. Blood.

[47] Robert P., Canault M., Farnarier C., Nurden A., Grosdidier C., Barlogis V., Bongrand P., Pierres A., Chambost H., Alessi M.C. (2011). A novel leukocyte adhesion deficiency III variant: Kindlin-3 deficiency results in integrin- and nonintegrin-related defects in different steps of leukocyte adhesion. J. Immunol..

[48] Shahid S., Zaidi S., Ahmed S., Siddiqui S., Abid A., Malik S., Shamsi T. (2019). A Novel Nonsense Mutation in FERMT3 Causes LAD-III in a Pakistani Family. Front. Genet..

[49] Mazaud-Guittot S., Meugnier E., Pesenti S., Wu X., Vidal H., Gow A., le Magueresse-Battistoni B. (2010). Claudin 11 deficiency in mice results in loss of the Sertoli cell epithelial phenotype in the testis. Biol. Reprod..

[50] Wu X., Peppi M., Vengalil M.J., Maheras K.J., Southwood C.M., Bradley M., Gow A. (2012). Transgene-mediated rescue of spermatogenesis in Cldn11-null mice. Biol. Reprod..

[51] Cheng C.Y., Mruk D.D. (2002). Cell junction dynamics in the testis: Sertoli-germ cell interactions and male contraceptive development. Physiol. Rev..

[52] Wang N., Verna L., Chen N.G., Chen J., Li H., Forman B.M., Stemerman M.B. (2002). Constitutive activation of peroxisome proliferator-activated receptor-gamma suppresses pro-inflammatory adhesion molecules in human vascular endothelial cells. J. Biol. Chem..

[53] Shen B., Chu E.S., Zhao G., Man K., Wu C.W., Cheng J.T., Li G., Nie Y., Lo C.M., Teoh N. (2012). PPARgamma inhibits hepatocellular carcinoma metastases in vitro and in mice. Br. J. Cancer.

[54] Li X.F., Sun Y.Y., Bao J., Chen X., Li Y.H., Yang Y., Zhang L., Huang C., Wu B.M., Meng X.M. (2017). Functional role of PPAR-γ on the proliferation and migration of fibroblast-like synoviocytes in rheumatoid arthritis. Sci. Rep..

[55] Goetze S., Xi X.P., Kawano H., Gotlibowski T., Fleck E., Hsueh W.A., Law R.E. (1999). PPAR gamma-ligands inhibit migration mediated by multiple chemoattractants in vascular smooth muscle cells. J. Cardiovasc. Pharmacol..

[56] Hase T., Yoshimura R., Mitsuhashi M., Segawa Y., Kawahito Y., Wada S., Nakatani T., Sano H. (2002). Expression of peroxisome proliferator-activated receptors in human testicular cancer and growth inhibition by its agonists. Urology.

[57] Bressler R.S. (1978). Hormonal control of postnatal maturation of the seminiferous cord. Ann. Biol. Animal Biochim. Biophys..

[58] Kosco M.S., Loseth K.J., Crabo B.G. (1989). Development of the seminiferous tubules after neonatal hemicastration in the boar. J. Reprod. Fertil..

[59] Zhao M., Chen Y., Ding G., Xu Y., Bai M., Zhang Y., Jia Z., Huang S., Zhang A. (2016). Renal tubular epithelium-targeted peroxisome proliferator-activated receptor-γ maintains the epithelial phenotype and antagonizes renal fibrogenesis. Oncotarget.

[60] Oyama T., Harigaya K., Sasaki N., Okamura Y., Kokubo H., Saga Y., Hozumi K., Suganami A., Tamura Y., Nagase T. (2011). Mastermind-like 1 (MamL1) and mastermind-like 3 (MamL3) are essential for Notch signaling in vivo. Development.

[61] Borggrefe T., Oswald F. (2009). The Notch signaling pathway: Transcriptional regulation at Notch target genes. Cell Mol. Life Sci..

[62] Dirami G., Ravindranath N., Achi M.V., Dym M. (2001). Expression of Notch pathway components in spermatogonia and Sertoli cells of neonatal mice. J. Androl..

[63] Defalco T., Saraswathula A., Briot A., Iruela-Arispe M.L., Capel B. (2013). Testosterone levels influence mouse fetal Leydig cell progenitors through notch signaling. Biol. Reprod..

[64] Kamińska A., Marek S., Pardyak L., Brzoskwinia M., Pawlicki P., Bilińska B., Hejmej A. (2020). Disruption of androgen signaling during puberty affects Notch pathway in rat seminiferous epithelium. Reprod. Biol. Endocrinol..

[65] Soares R., Balogh G., Guo S., Gärtner F., Russo J., Schmitt F. (2004). Evidence for the notch signaling pathway on the role of estrogen in angiogenesis. Mol. Endocrinol..

[66] Murta D., Batista M., Silva E., Trindade A., Henrique D., Duarte A., Lopes-da-Costa L. (2013). Dynamics of Notch pathway expression during mouse testis post-natal development and along the spermatogenic cycle. PLoS ONE.

[67] Hasegawa K., Okamura Y., Saga Y. (2012). Notch signaling in Sertoli cells regulates cyclical gene expression of Hes1 but is dispensable for mouse spermatogenesis. Mol. Cell Biol..

